# Factors associated with the use and reuse of face masks among Brazilian individuals during the COVID-19 pandemic^*^

**DOI:** 10.1590/1518-8345.4604.3360

**Published:** 2020-09-07

**Authors:** Fernanda Maria Vieira Pereira-Ávila, Simon Ching Lam, Fernanda Garcia Bezerra Góes, Elucir Gir, Natalia Maria Vieira Pereira-Caldeira, Sheila Araújo Teles, Karla Antonieta Amorim Caetano, Maithê de Carvalho e Lemos Goulart, Thamara Rodrigues Bazilio, Ana Cristina de Oliveira e Silva

**Affiliations:** 1 Universidade Federal Fluminense, Departamento de Enfermagem Rio das Ostras, Rio das Ostras, RJ, Brazil.; 2 Hong Kong Polythechnic University, School of Nursing, Hong Kong, China.; 3 Universidade de São Paulo, Escola de Enfermagem de Ribeirão Preto, PAHO/WHO Colaborating Centre at the Nursing Research Development, Ribeirão Preto, SP, Brazil.; 4 Universidade Federal de Goiás, Faculdade de Enfermagem, Goiânia, GO, Brazil.; 5 Universidade Federal da Paraíba, Departamento de Enfermagem Clinica, João Pessoa, PB, Brazil.

**Keywords:** Masks, Pandemic, Coronavirus, Infections by Coronavirus, COVID-19, Reuse, Mascarillas, Pandemias, Coronavirus, Infecciones por coronavirus, COVID-19, Reutilización, Máscaras, Pandemias, Coronavirus, Infecções por Coronavírus, COVID-19, Reutilização

## Abstract

**Objective:**

to identify the factors associated with the use and reuse of masks among Brazilian individuals in the context of the COVID-19 pandemic.

**Method:**

cross-sectional study conducted in the five Brazilian regions, among adult individuals, via an electronic form disseminated in social media, addressing general information and the use of masks. Bivariate analysis and binary logistic regression were used to identify the factors associated with the use and reuse of masks.

**Results:**

3,981 (100%) individuals participated in the study. In total, 95.5% (CI 95%: 94.8-96.1) reported using masks. Fabric masks were more frequently reported (72.7%; CI 95%: 71.3-74.1), followed by surgical masks (27.8%; CI 95%: 26.5-29.2). The percentage of reuse was 71.1% (CI 95%: 69.7-72.5). Most (55.8%; CI 95%: 51.7-60.0) of those exclusively wearing surgical masks reported its reuse. Being a woman and having had contact with individuals presenting respiratory symptoms increased the likelihood of wearing masks (p≤0.001). Additionally, being a woman decreased the likelihood of reusing surgical masks (p≤0.001).

**Conclusion:**

virtually all the participants reported the use of masks, most frequently fabric masks. The findings draw attention to a risky practice, that of reusing surgical and paper masks. Therefore, guidelines, public policies, and educational strategies are needed to promote the correct use of masks to control and prevent COVID-19.

## Abstract (es)

**Objetivo:**

identificar los factores asociados con el uso y la reutilización de máscaras entre brasileños durante la pandemia del COVID-19.

**Método:**

estudio transversal desarrollado en las cinco regiones brasileñas, entre adultos, aplicándose un formulario electrónico a través de las redes sociales, que contiene información general y relativo al uso de máscaras. Se utilizaron análisis bivariados y regresiones logísticas binarias para identificar los factores asociados con el uso y la reutilización.

**Resultados:**

3.981 (100%) personas participaron en el estudio. El uso de máscaras totalizó 95.5% (IC 95%: 94.8-96.1), particularmente las de tejido (72.7%; IC 95%: 71.3-74.1) y la quirúrgicas (27.8%; IC 95%: 26.5-29.2). El porcentaje de reutilización fue del 71,1% (IC 95%: 69,7-72,5). La mayoría (55.8%; IC 95%: 51.7-60.0) que usa exclusivamente una máscara quirúrgica la reutiliza. El género femenino y el contacto previo con personas con síntomas respiratorios aumentaron las posibilidades de uso (p≤0.001). Sin embargo, el sexo femenino disminuyó la posibilidad de reutilizar la máscara quirúrgica (p≤0.001).

**Conclusión:**

casi todos los participantes informaron haber usado máscaras, siendo aquella de tejido más frecuente. Los hallazgos llaman la atención sobre una práctica arriesgada, la reutilización de la máscara quirúrgica y de papel. Las directrices, políticas públicas y estrategias educativas son necesarias para el desarrollo de prácticas asertivas en el control y la prevención del COVID-19.

## Abstract (pt)

**Objetivo:**

identificar os fatores associados à utilização e à reutilização de máscaras entre brasileiros durante a pandemia da COVID-19.

**Método:**

estudo transversal desenvolvido nas cinco regiões brasileiras, entre adultos, via formulário eletrônico por meio de mídias sociais, contendo informações gerais e referentes à utilização de máscaras. Foram utilizadas análises bivariadas e regressões logísticas binárias para identificar fatores associados à utilização e à reutilização.

**Resultados:**

participaram do estudo 3.981 (100%) pessoas. A utilização de máscaras totalizou 95,5%, (IC 95%: 94,8-96,1), com destaque para as de tecido (72,7%; IC 95%: 71,3-74,1) e as cirúrgicas (27,8%; IC 95%: 26,5-29,2). A porcentagem de reutilização foi de 71,1% (IC 95%: 69,7-72,5). A maioria (55,8%; IC 95%: 51,7-60,0) que usa, exclusivamente, máscara cirúrgica reutiliza-a. O sexo feminino e o contato prévio com pessoas com sintomas respiratórios aumentaram as chances de utilização (p≤0,001). Contudo, o sexo feminino diminuiu a possibilidade de reutilização da máscara cirúrgica (p≤0,001).

**Conclusão:**

a quase totalidade dos participantes relatou utilizar máscaras, sendo mais recorrente a de tecido. Os achados chamam atenção para uma prática de risco, a reutilização da máscara cirúrgica e de papel. Diretrizes, políticas públicas e estratégias educativas são necessárias para o desenvolvimento de práticas assertivas no controle e prevenção da COVID-19.

## Introduction

The COVID-19 pandemic, a disease caused by the novel human coronavirus (SARS-CoV-2), has affected people in all continents, with 6,912,751 cases confirmed up to June 8^th^, 2020, along with 400,469 deaths^(1)^. Brazil ranks third in the world in the number of deaths; more than 680,456 cases and 36,151 deaths were confirmed up to the same date in the country^(1-2)^.

Symptoms among those infected with SARS-CoV-2 range from no symptoms up to severe cases of pulmonary disease. The incubation period that involves the onset of the first symptoms may range from two to 14 days after the infection, though some people do not present any symptoms^(3)^.

Note that, even if asymptomatic, individuals infected with SARS-CoV-2 have the potential to transmit the virus^(3-4)^. Transmission mainly occurs from person to person through respiratory droplets, when an individual coughs, sneezes or speaks near other people, while contaminated surfaces or objects may also be sources of transmission^(5)^.

Therefore, the general population is advised to wear face masks as a mechanical barrier to prevent the dispersion of droplets, in addition to frequent hand sanitization using soap and water or alcohol-based solutions^(3)^. Hence, to self-protect against diseases and also prevent the transmission of pathogens between healthy and sick individuals, masks have been used as a popular public health intervention^(6)^.

In Brazil, given a shortage of personal protective equipment (PPE), especially surgical masks, the Ministry of Health advised the population, through an informative note published on April 2^nd^, 2020, to make and wear their homemade masks. It also advises that masks made of vacuum bags, cotton, or antimicrobial pillowcases ensure an effective physical barrier, provided they are properly designed and sanitized^(7)^.

Note that to ensure masks are effective for what they are proposed, that is, to impede the dissemination of expelled droplets, masks need to completely cover mouth and nose and adjust well to the face, without side clearances. Homemade masks are recommended to be sanitized before reuse^(7)^. Surgical masks are disposable and should not be sanitized because they lose their filtering capacity. Similarly, N95 respirators should not be washed and the number of times it may be reused is informed by manufacturers^(8)^.

How frequently the population wears masks increases with the onset of local epidemics. It is, however, necessary to better investigate factors such as the duration of protection and how this piece of PPE is used and reused, considering that inappropriate use, especially of disposable masks, may compromise its protective effect and even raise the risk of infection^(9)^.

In this sense, considering that the use of masks is not a common practice in the population in general, in times of pandemic, this measure has been strongly encouraged and represents a new reality experienced by the Brazilian people. Therefore, identify how people have used and reused this protective piece of equipment is extremely important to discuss the potential effects of not wearing the different types of masks or wearing them inappropriately in the face of the COVID-19 pandemic. Hence, this study’s objective was to identify the factors associated with the use and reuse of masks among Brazilian individuals during the COVID-19 pandemic.

## Method

This cross-sectional study was conducted between April 16^th^ and 27^th^ in the North, Northeast, Southeast, Midwest, and South of Brazil. This study is part of a multinational project addressing the practice of reusing masks in the population in general during the COVID-19 pandemic in Brazil and is part of an international study “Practice of face mask use among general public during the outbreak of COVID-19: a multi-country cross-sectional study”, in partnership with Squina International Centre for Infection Control, Hong Kong Polytechnic University.

According to data made available by the Brazilian Institute of Geography and Statistics (*Instituto Brasileiro de Geografia e Estatística* - IBGE) concerning the 4^th^ trimester of 2019, the population of Brazilian individuals aged over 18 years old is approximately 159,095,000. A representative sample of this population group was used. A finite population sample was calculated with a confidence interval of 98%, sampling error of 2%, prevalence of 50%, and test power of 80% and a minimum sample of 3,393 individuals was found. The inclusion criterion was being 18 years old or older, while the exclusion criterion was being a foreigner living in Brazil or a health worker.

Data were collected through social media such as Facebook, Twitter, Instagram, Whatsapp, and e-mail. A form composed of two parts was applied: 1- General information (sex, age, state of residence, educational level, marital status, and contact with individuals with respiratory symptoms); 2- Information concerning the use of masks: type of mask used (paper mask, fabric mask, surgical mask, active carbon mask, N95, or others), frequency of reuse (number of times a mask is reused). Considering that it is a multinational study, the questions were translated from English to Portuguese by two independent translators and were content validated by a panel composed of five experts in the subject. No changes were suggested. A pilot test was conducted prior to the data collection with 20 individuals conveniently selected from five different Brazilian regions to verify the instrument’s sensitiveness and applicability. Only adaptations in terms of the form’s online presentation and layout were suggested and implemented. The instrument was converted to the online format using Google Forms.

Invitation to participate in the study was sent through messages disseminated in social media, containing a link to the survey. The researchers also published the link to access the instrument in their social media profiles. By accessing the link, the Internet user was directed to the Google platform with access to the form. A free and informed consent form was presented on the initial page and participants only accessed the instrument of data collection after providing their consent.

The outcome (dependent) variables were mask use and surgical mask reuse. The independent variables for both were: sex, age group, educational level, marital status, and contact with people presenting respiratory symptoms.

Data were exported from Google Forms directly to a spreadsheet in Microsoft Office Excel^®^ and later to IBM^®^ SPSS, version 20.0. Descriptive statistical analysis included absolute and relative frequencies, to characterize the participants and data concerning the use and reuse of masks, in addition to central tendency (mean, median, maximum and minimum) and dispersion measures (standard deviation) for age.

Prevalence concerning the use and reuse of masks was estimated considering a confidence interval of 95% (CI 95%). The Chi-square test was adopted to analyze the association between the outcome and independent variables. Associations with p<0.20 were verified using binary logistic regression considering the dependent variables were dichotomous, the probabilities concerning the use of face masks and reuse of surgical masks in the population were verified using Odds Ratio (OR) and its respective confidence intervals of 95%. Associations were considered statistically significant when p<0.05.

The project was submitted to and approved by the National Committee of Ethics Research (*Comissão Nacional de Ética em Pesquisa* - CONEP) (CAAE: 30572120.0.0000.0008; Opinion 3.971.512). All ethical aspects were complied with in Brazil according to Resolutions 466/2012 and 510/2016 of the National Health Council. Financial support: *Conselho Nacional de Desenvolvimento Científico e Tecnológico* (CNPq), Brazil, grant #401371/2020-4.

## Results

In total, 3,981 (100%) individuals participated in the study. Regarding sociodemographic characteristics, the regions with the largest number of participants were the Southeast with 1,874 (47.1%) and Northeast with 1,348 (33.9%), followed by the Midwest with 320 (8.0%), North with 265 (6.7%), and South with 174 (4.4%). Most, 2,893 (72.7%) were women, 2,057 (51.7%) were married, aged 40 years old on average (SD=13.9); minimum 18 and maximum 86 years old. Concerning the educational level, most participants, 3,280 (82.4%), reported holding a college education degree. Most, 2,622 (65.9%), reported no close contact with individuals with respiratory symptoms; 1,359 (34.1%) mentioned at least one contact, 224 (5.6%) of whom had daily and continuous contact with people presenting respiratory symptoms (Table 1).

**Table 1:** – Characterization of the participants (n=3,981) according to sex, age group, educational level, marital status, and contact with people presenting respiratory symptoms, according to Brazilian region. Brazil, 2020

Variables	Brazilian regions				
	North	Northeast	Southeast	South	Midwest
	n (%)	n (%)	n (%)	n (%)	n (%)
Sex					
^ ^Male	79 (7.3)	402 (36.9)	487 (44.8)	36 (3.3)	84 (7.7)
Female	186 (6.4)	946 (32.7)	1387 (47.9)	138 (4.8)	236 (8.2)
Age group (years)					
18 to 24	69 (10.8)	298 (46.7)	197 (30.9)	18 (2.8)	56 (8.8)
25 to 39	120 (8.1)	560 (37.9)	611 (41.3)	70 (4.7)	117 (7.9)
40 to 59	66 (4.5)	417 (28.6)	802 (55.0)	63 (4.3)	111 (7.6)
60 or more	10 (2.5)	73 (18.0)	264 (65.0)	23 (5.7)	36 (8.9)
Educational Level					
Middle School	2 (5.9)	11 (32.4)	17 (50.0)	1 (2.9)	3 (8.8)
High School	54 (8.1)	246 (36.9)	304 (45.6)	17 (2.5)	46 (6.9)
College	209 (6.4)	1091 (33.3)	1553 (47.3)	156 (4.8)	271 (8.3)
Marital Status					
Single	148 (9.9)	581 (38.7)	587 (39.1)	63 (4.2)	122 (8.1)
Married	97 (4.7)	652 (31.7)	1052 (51.1)	94 (4.6)	162 (7.9)
Separated/Divorced	19 (5.4)	100 (28.5)	186 (53.0)	15 (4.3)	31 (8.8)
Widowed	1 (1.4)	15 (20.8)	49 (68.1)	2 (2.8)	5 (6.9)
Contact w/ people w/ respiratory problems					
Never	156 (5.9)	918 (35.0)	1238 (47.2)	109 (4.2)	201 (7.7)
Rarely	63 (7.9)	246 (30.7)	380 (47.4)	45 (5.6)	68 (8.5)
Last month	5 (8.2)	26 (42.6)	24 (39.3)	1 (1.6)	5 (8.2)
Last week	15 (10.1)	43 (28.9)	67 (45.0)	6 (4.0)	18 (12.1)
Last day	8 (6.5)	46 (37.4)	54 (43.9)	6 (4.9)	9 (7.3)
Daily and continuous	18 (8.0)	69 (30.8)	111 (49.6)	7 (3.1)	19 (8.5)

Most of the participants, 3,803 (95.5%; CI 95%: 94.8-96.1) reported the use of masks; only 178 individuals (4.5%; CI 95%: 3.9-5.1) reporting not wearing masks. When the frequency is verified *per* region, the region with the highest rate of use was the South with 172 (98.9%; CI 95%: 96.7-99.8), followed by the North with 259 (97.7%; CI 95%: 95.3-99.1), Northeast with 1,290 (95.7%; CI 95%: 94.5-96.7), Southeast with 1,779 (94.9%; CI 95%: 93.9-95.9) and the Midwest with 303 (94.7%; CI 95%: 91.8-96.8).

Regarding the type of mask used, the most frequently reported type was fabric/cotton homemade masks, used exclusively or in combination with other masks, 2,895 (72.7%; CI 95%: 71.3-74.1), followed by surgical masks, reported by 1,108 (27.8%; CI 95%: 26.5-29.2) individuals, N95 respirators reported by 335 (8.4%; CI 95%: 7.6-9.3), paper or gauze reported by 293 (7.4%; CI 95%: 6.6-8.2), active carbon by 38 (1.0%; CI 95%: 0.7-1.3), industrial masks by 14 (0.4%; CI 95%: 0.2-0.6), and other types of masks were reported by 10 (0.3%; CI 95%: 0.1-0.4) participants.

Note that most of the 3,803 (100%) individuals who reported the use of masks, 3,002 (78.9%; CI 95%: 77.6-80.2), reported wearing only one type of mask, followed by 716 (18.8%; CI 95%: 17.6-20.1) who reported two types of masks; 80 (2.1%; CI 95%: 1.7-2.6) individuals reported three types; three individuals (0.0%; CI 95%: 0.0-0.2) reported wearing four different types of masks; and two individuals (0.0%; CI 95%: 0.0-0.2) reported five types of masks. The combination of masks most frequently reported was fabric/cotton homemade masks in combination with surgical masks, 371 (9.7%; CI 95%: 8.8-10.7).

The variables that presented p<0.20 in the association between variables were: sex (p=0.000), age (p=0.084), educational level (p=0.047), and contact with individuals presenting respiratory symptoms (p=0.001) (Table 2). These variables were included in the binary logistic regression model for the outcome variable “use of mask”.

**Table 2:** – Association between the use of mask and demographic variables and contact with people with respiratory symptoms (n=3,981). Brazil, 2020

Use of Masks			
Variables	No	Yes	P-value
	n (%; CI 95%)^*^	n (%; CI 95%)^*^	
**Sex**			
Male	74 (6.8; 5.4-8.4)	1014 (93.2; 91.6-94.6)	**0.000**
Female	104 (3.6; 3.0-4.3)	2789 (96.4; 95.6-97.0)	
Age group (years)			
18 to 24	35 (5.5; 3.9-7.5)	603 (94.5; 92.5-96.1)	0.084
25 to 39	76 (5.1; 4.1-6.3)	1402 (94.9; 93.6-95.9)	
40 to 59	54 (3.7; 2.8-4.8)	1405 (96.3; 95.2-97.1)	
60 old or older	13 (3.2; 1.8-5.3)	393 (96.8; 94.7-98.2)	
Educational level			
Middle school	3 (8.8: 2.3-22.2)	31 (91.2; 77.8-97.7)	**0.047**
High School	40 (6.0; 4.4-8.0)	627 (94.0; 92.0-95.6)	
College	135 (4.1; 3.5-4.8)	3145 (95.9; 95.1-96.5)	
Marital status			
Married	81 (4.2; 3.4-5.2)	1843 (95.8; 94.8-96.6)	0.440
Not married	97 (4.7; 3.9-5.7)	1960 (95.3; 94.3-96.1)	
Contact w/ people w/ respiratory problems			
No	137 (5.2; 4.4-6.1)	2485 (94.8; 93.9-95.6)	**0.001**
Yes	41 (3.0; 2.2-4.0)	1318 (97.0; 96.0-97.8)	

^*^CI = Confidence interval

Among the 3,803 (100%) who reported the use of masks, 2,832 (74.5%; CI 95%: 73.1-75.8) reported reusing masks, while 1,149 (30.2%; CI 95%: 28.8-31.7) did not reuse masks. Among the 2,832 (100%) who reuse masks, 947 (33.4%; CI 95%: 31.7-35.2) reported reusing 1-2 times; 717 (25.3%; CI 95%: 23.7-27.0) reported masks were used 3-4 times; 661 (23.3%; CI 95%: 21.8-24.9) reported reusing masks more than 7 times; and 507 (17.9%; CI 95%: 16.5-19.3) reported reusing masks 5-6 times (Table 3).

**Table 3:** – Distribution of types of masks according to reuse (n=3,803). Brazil, 2020

Types of Masks	Reuse of face masks				
	Never	1-2 times	3-4 times	5-6 times	More than 7
	n (%; CI 95%)^*^	n (%; CI 95%)^*^	n (%; CI 95%)^*^	n (%; CI 95%)^*^	n (%; CI 95%)^*^
Paper/gauze	71 (54.2; 45.6-62.6)	24 (18.3; 12.4-25.6)	23 (17.6; 11.7-24.8)	05 (3.8; 1.4-8.2)	08 (6.1; 2.9-11.3)
Fabric/cotton	467 (21.7; 20.0-23.5)	513 (23.8; 22.1-25.7)	371 (17.2; 15.7-18.9)	318 (14.8; 13.3-16.3)	483 (22.4; 20.7-24.2)
Surgical	243 (44.1; 40.0-48.3)	150 (27.2; 23.6-31.1)	91 (16.5; 13.6-19.8)	44 (8.0; 5.9-10.5)	23 (4.2; 2.7-6.1)
Active carbon	01 (14.3; 0.7-53.0)	02 (28.6; 5.1-67.0)	02 (28.6; 5.1-67.0)	02 (28.6; 5.1-67.0)	0 (0.0; 0.0-34.8)
N95	10 (7.0; 3.6-12.2)	22 (15.5; 10.2-22.1)	41 (28.9; 21.9-36.7)	36 (25.4; 18.7-33.0)	33 (23.2; 16.8-30.7)
Industrial	01 (11.1; 0.5-43.9)	01 (11.1; 0.5-43.9)	01 (11.1; 0.5-43.9)	0 (0.0; 0.0-28.3)	06 (66.7; 33.2-90.7)
Others	05 (50.0; 21.2-78.8)	2 (20.0; 3.5-52.0)	02 (20.0; 3.5-52.0)	0 (0.0; 0.0-25.9)	01 (10.0; 0.5-40.3)
Combination of masks	188 (23.5; 20.6-26.5)	226 (28.2; 25.1-31.4)	185 (23.1; 8.6-12.9)	98 (12.2; 10.1-14.6)	104 (13.0; 10.8-15.4)

^*^CI = Confidence interval

Note that most of the 551 (100%) participants who report exclusively wearing surgical masks, 308 (55.8%; CI 95%: 51.7-60.0) reuse masks according to the following frequencies: 150 (27.2%) rarely/1-2 times reuse masks, 91 (16.5%) reuse sometimes/3-4 times, 44 (8.0%) frequently/5-6 times, and 23 (4.2%) participants reuse always/7 times or more. Of those, 131 (100%) who wear only paper or gauze masks, 60 (45.8%; CI 95%: 37.4-54.4) participants reuse according to the following frequencies: 24 (18.3%) reported using masks rarely/1-2 times; 23 (17.6%) sometimes/3-4 times, 8 (6.1%) reported always/7 times or more, and 5 participants (3.8%) reported reusing masks frequently/5-6 times. Among the 2,152 (100%) who exclusively wear fabric/cotton masks, 1,685 (78.3%; CI 95%: 76.5-80.1) reuse according to the following frequencies: 513 (23.8%) reported rarely/1-2 times; 483 (22.4%) reported always/7 times or more; 371 (17.2%) reported sometimes/3-4 times, and 318 (14.8%) reported frequently/5-6 times. The confidence intervals concerning the prevalence of reuse according to types of masks are presented in Table 3.

In the association between variations concerning the reuse of surgical masks, sex (p=0.001) was the variable that presented p<0.20 and was thus included in the binary logistic regression model for the outcome variable “reuse of mask” (Table 4).

**Table 4:** – Association between the reuse of surgical masks and demographic variables and contact with people with respiratory symptoms (N=551). Brazil, 2020

	Reuse of surgical masks		
Variables	No	Yes	P-value
	n (%; CI 95%)^*^	n (%; CI 95%)^*^	
Sex			
Male	60 (33.9; 27.2;41.1)	117 (66.1; 58.9-73.0)	**0.001**
Female	183 (48.9; 43.9-54.0)	191 (51.1; 46.0-56.1)	
Age group (years)			
18 to 24	53 (51.0; 41.4-60.5)	51 (49.0; 39.5-58.6)	0.279
25 to 39	87 (43.9; 37.1-50.9)	111 (56.1; 49.1-62.9)	
40 to 59	86 (43.0; 36.3-49.9)	114 (57.0; 50.1-63.7)	
60 old or older	17 (34.7; 22.4-48.7)	32 (65.3; 51.3-77.6)	
Educational level			
Middle school	0 (0.0; 0.0-63.2)	3 (100.0; 36.9-100.0)	0.303
High School	51 (44.0; 35.1-53.1)	65 (56.0; 46.9-64.9)	
College	192 (44.4; 39.8-49.1)	240 (55.6; 50.8-60.3)	
Marital status			
Married	123 (46.8; 40.8-52.8)	140 (53.2; 47.2-59.4)	0.315
Not married	120 (41.7; 36.1-47.4)	168 (58.3; 52.6-63.9)	
Contact w/ people w/ respiratory problems			
No	154 (45.0; 39.8-50.3)	188 (55.0; 49.7-60.2)	0.575
Yes	89 (42.6; 36.0-49.4)	120 (57.4; 50.6-64.0)	

^*^CI = Confidence interval

After the binary logistic regression, being a woman (OR=2.02; CI 95%: 1.48-2.75; p=0.000) continued as a factor associated with the more frequent use of masks, presenting approximately twice the likelihood of wearing masks, while having contact with people presenting respiratory symptoms (OR=1.80; CI: 1.26-2.58; p=0.001) increased approximately 1.8 times the likelihood of wearing masks. Being a woman (OR=0.53; CI: 0.37-0.78; p=0.001) also remained a factor associated with the reuse of surgical masks, though, the reuse of surgical masks was two times less likely among women than in the population (Table 5).

**Table 5:** – Odds Ratio according to binary logistic regression for the use and reuse of surgical masks. Brazil, 2020

Variables	Use and Reuse of Masks	P-value
Use of Mask OR^*^ (CI 95%)^†^		
Female sex	2.02 (1.48-2.75)	**0.000**
Contact w/ people w/ respiratory symptoms	1.80 (1.26-2.58)	**0.001**
Reuse of Surgical Mask OR^*^ (CI 95%)^†^		
Female sex	0.53 (0.37-0.78)	**0.001**

^*^OR = Odds ratio; ^†^CI = Confidence interval

## Discussion

This study’s findings reveal the pattern of use and reuse of masks in the Brazilian population. Virtually all the participants reported the use of this piece of PPE and fabric masks were the most frequently reported. As the COVID-19 pandemic increasingly aggravated worldwide, the use of masks has been recommended to the population as a non-pharmacological intervention that plays a vital role in controlling the spread of the disease, mainly because the dissemination of the SARS-CoV-2 from human to human mainly occurs through respiratory droplets^(10-11)^.

The recommendation for the population to wear homemade masks during epidemics of respiratory infectious diseases such as the Severe Acute Respiratory Syndrome (SARS) and COVID-19, has been successful as a public health intervention at the global level. In China, for instance, this practice has been efficacious in combination with other protective strategies to prevent the dissemination of viruses causing these two diseases^(12)^.

The populations of many countries in all the continents have adhered to the use of masks due to the rapid dissemination of the SARS-CoV-2. People in China, including Hong Kong, and Japan, Thailand, and South Korea, have used different types of masks. The growth of cases of COVID-19 in the Czech Republic, where the government has made its use mandatory, has been slower than in other European countries^(13)^.

On April 2^nd^, 2020^(7)^, the Brazilian Ministry of Health recommended the population at a national level to start wearing masks. The adoption of this recommendation is confirmed in a study conducted in the second half of April of the same year, which reports that the prevalence of the use of masks was almost absolute in all Brazilian regions; the South presented the highest prevalence. Being a woman doubles the likelihood of wearing masks. One study conducted in China, addressing 10,304 participants during the period of the COVID-19 pandemic, also reports greater adherence to protective measures among women, indicating that sex influences adherence to official recommendations related to health^(14)^.

In this study, the likelihood of wearing masks almost doubled among those who had contact with individuals with respiratory symptoms, which is in agreement with the World Health Organization’s recommendation to those with a confirmed diagnosis or suspected infection and their respective caregivers to wear masks^(13)^. One Chinese study, however, verified that this use, regardless of the presence of respiratory symptoms, was associated with lower levels of anxiety and depression, that is, the use of masks has potential psychological benefits, giving a sense of safety^(15)^. Even though masks promote a sense of safety, despite having contact with asymptomatic individuals or those with symptoms, the use of masks cannot be the only measure adopted at the expense of other essential protective measures to prevent COVID-19. Social distancing and hand sanitation with soap and water, or with an antiseptic solution of at least 60% alcohol, are highlighted^(3)^.

Another aspect to take into account is the participants’ educational level, as most reported a higher education degree, suggesting they had a better understanding of the importance of wearing masks to prevent the COVID-19. This finding corroborates with a literature review addressing the use of masks to prevent respiratory infections, which revealed that higher education was positively associated with the practice^(16)^, reinforcing the need for educational strategies directed to the entire population, especially those with fewer years of schooling.

Regarding the type of masks, those made of fabric predominated in this population, emerging as an alternative in the face of a shortage of surgical masks. Even though fabric masks are not as effective as those recommended for hospital use, adherence to fabric masks reduces the risk of disseminating respiratory infections. Additionally, they are particularly popular in developing countries given their availability, low cost, and the possibility to be reused after washing^(17)^. As current results show, the use of this type of mask among Brazilian individuals has been prevalent during the COVID-19 pandemic.

The reuse of this piece of PPE was a practice mentioned by a large portion of the individuals addressed in this study. The reuse of masks requires attention and should be better discussed with the population adopting this practice. Fabric masks need to be washed after being used. The circumstances in which individuals consider it necessary to wash masks were not addressed in this study, but it became apparent that a good portion of the participants reused masks, at least five times. Washing, drying, and stretching gradually increase the porosity of fabrics and therefore, impair filtration efficiency, as a study conducted in Nepal shows^(17)^. Therefore, investment is needed, especially on the part of public policymakers, to provide educational guidelines on how to reuse masks to the population in general.

The study shows that, even though surgical masks are recommended to be discarded after use, reuse reports were considerably high. Surgical masks are disposable and should not be cleaned or disinfected for later use as they lose their filtration capacity when wet^(8)^. Additionally, more than half of the participants who reported the exclusive use of surgical masks reuse them more than once, revealing a risky practice that demands the immediate intensification of public intervention, using educational strategies to ensure the correct use of this piece of protective equipment.

Researchers have tested alternatives to decontaminate masks, including surgical masks, however, some of these alternatives may damage the masks’ blocking structure through physical or chemical action, or incompletely inactivate pathogens. Moreover, these alternatives may not be available to the population in general as they require specific instruments or material. Additionally, further studies are needed to assess appropriate measures to decontaminate N95 respirators and surgical masks^(18)^; studies have not reported conclusive evidence. Therefore, the reuse of surgical masks is not recommended to the population without an effective possibility to safely decontaminate them. In fact, the reuse of surgical masks may even increase the risk of contamination by COVID-19.

Another noteworthy finding in this study is the considerable number of people who use, and reuse, paper or gauze masks as respiratory protection, even though, this type of masks have not been recommended in Brazil. Another factor is that the reuse of masks is less frequent among women than among men, reinforcing that sex is associated with adherence or lack of adherence to protective practices^(14)^. Hence, educational actions are necessary to address preventive measures in the context of the COVID-19 among the male population.

This study only addressed the use and reuse of masks in the Brazilian population, however, it is important to stress that masks must be adopted together with other preventive measures. This research draws attention to two concerning facts: the inappropriate use of masks and/or the possibility that people become careless with other preventive measures when wearing masks. Therefore, the set of measures that can decrease the spread of SARS-CoV-2 and flatten the pandemic curve, include social distancing, respiratory etiquette, and handwashing, together with the appropriate use of masks^(11)^. These are situations that should be carefully assessed by health managers in Brazil to devise more assertive plans and protocols.

The current COVID-19 pandemic remains severe worldwide and is an international source of concern, considering that it is a disease with high contagious potential, so the importance of sensitizing the population, reinforcing control measures^(19)^.

This study’s limitations include the fact that digital illiterates were excluded, the impossibility to clarify doubts when participants did not understand a question and lack of knowledge regarding the circumstances in which the form was completed.

## Conclusion

The results show that most of this study’s participants reported the use of masks and that fabric masks were more frequently reported. Being a woman and prior contact with individuals with respiratory symptoms were associated with the use of masks, increasing its likelihood. Being a woman was also associated with the reuse of surgical masks though, as women are twice less likely to reuse this type of mask in the population.

This study’s findings draw attention to a risk practice, that of reusing paper and surgical masks, which increases the chance of transmission given inefficacious respiratory protection. In this sense, this study presents important contributions in the health field as it provides new scientific evidence regarding the use and reuse of masks, which can be used to support the establishment of guidelines, public policies and educational strategies to promote the adoption of correct practices to control and prevent the COVID-19 in the Brazilian territory.
